# Objective evaluation of nonsurgical treatment of prominent ears: A systematic review

**DOI:** 10.1016/j.jpra.2023.07.002

**Published:** 2023-07-22

**Authors:** Yangyang Lin, Elsa M. Ronde, Hashir A. Butt, F.S. van Etten-Jamaludin, Corstiaan C. Breugem

**Affiliations:** a Department of Plastic, Reconstructive and Hand Surgery, Amsterdam UMC, Amsterdam Medical Centre, Amsterdam, The Netherlands; b Bachelor of Science in Medicine, Amsterdam UMC, location AMC, University of Amsterdam, The Netherlands; c Amsterdam UMC, University of Amsterdam, Research Support, Medical Library Academic Medical Center, Amsterdam, the Netherlands

**Keywords:** Prominent ears, Nonsurgical treatment, Splinting, Ear-well, Objective evaluation

## Abstract

**Background:**

The prominent ear is a type of congenital ear deformity that can be corrected by a variety of nonsurgical treatments, such as splinting and the taping method. However, there is no objective evaluation method that is universally accepted. The aim of this review is to evaluate objective measurement methods that are used in the available literature to analyze nonsurgical treatment of prominent ears.

**Methods:**

A systematic review was performed in the MEDLINE and Embase databases in December 2022 and updated on April 2023 according to Preferred Reporting Items for Systematics and Meta-Analyses (PRISMA) guideline. Any study using objective measurements (continuous variables such as distance and angle) to evaluate the effect of nonsurgical treatment of prominent ears was included. The Joanna Briggs Institute (JBI) critical appraisal for case series was used for quality assessment.

**Results:**

A total of 286 studies were screened for eligibility, of which five articles were eligible for inclusion. All of the included studies were case series. The helix mastoid distance (HMD) is the most commonly used parameter to measure treatment outcome. Pinna and cartilage stiffness, length, and width were also used, but without clear statistical relevance. HMD was classified into grading groups (i.e. good, moderate, and poor) to evaluate the treatment's effect.

**Conclusion:**

Based on the included studies, objective measurements are rarely used, and when used, they are largely heterogeneous. Although HMD was the most frequent measurement used, all studies used different definitions for the measurement and grouped subsequent outcomes differently. Automated algorithms, based on three-dimensional imaging, could be used for object measurements in the nonsurgical treatment of prominent ears.

## Introduction

Prominent ears are one of the most common congenital auricular deformities (CAD) affecting children.^1^ Having prominent ears can have a profound psychological impact on school-aged children and may lead to behavioral problems and poor school performance.^2^ Prominent ears are generally treated surgically (otoplasty), although nonsurgical treatments offer advantages by enabling treatment at a very early age and avoiding surgical complications.^3^ According to the Chinese expert consensus,^4^ nonsurgical treatment of CADs can be divided into surgical taping,^5^ wire-shaped splinting,^6^^,^^7^ clip-shaped splinting,^8^^,^^9^ and the EarWell system.^10^ All of these techniques use the malleability of newborn ear cartilage to change the shape of the ears. However, due to this approach, nonsurgical treatments generally have a long duration, and treatment outcomes may be unreliable.^6^

To our knowledge, there are no standardized tools to evaluate treatment outcomes of nonsurgical treatment of prominent ears, and outcomes are commonly evaluated subjectively.^6^ Objective evaluation techniques use measurement tools to obtain geometrically continuous quantitative data on the auricle. A typical example is the use of a ruler to compare the distance from the helix to the mastoid (HMD) pretreatment and posttreatment.^2^^,^^11^^,^^12^ As objective assessments can be conducted uniformly, objectively assessed outcomes should be reliable, enabling effective comparisons across and between treatment techniques. Furthermore, objective assessments may also be used for uniform diagnostics of prominent ears,^13^^,^^14^ and some clinical commissioning groups in the United Kingdom require ear measurements for insurance coverage.^15^

A previous systematic review summarized the outcomes of nonsurgical treatments of prominent ears. However, they did not focus solely on objectively evaluated outcomes.^6^ The aim of the current systematic review is to review the literature for objective measurements used in the nonsurgical treatment of prominent ears.

## Methods

We applied the Preferred Reporting Items for Systematics and Meta-Analyses (PRISMA) guideline for this systematic review,^16^ and registered it in PROSPERO (CRD42023393598).

### Search strategy and selection

A comprehensive literature search was conducted in MEDLINE (PubMed interface) and Embase (OVID interface) from inception until the 8^th^ of December 2022 and updated on the 24^th^ of April 2023 by a clinical librarian (FSEJ). The search comprised of a combination of keywords, including “prominent ears,” “congenital malformations,” “non-surgical,” “splinting,” and “molding.” To limit the possibility of missing relevant titles, we did not include terms on objective treatment outcomes as these may not be explicitly mentioned in titles or abstracts. A full version of the search strategy can be found in Supplementary file 1.

### Inclusion and exclusion criteria

Studies were included if (1) the patient group included patients with prominent ears, (2) a nonsurgical intervention was used (e.g. splinting), and (3) outcomes were measured objectively and quantitatively (i.e. by using an analog or digital measurement tool, and reported in e.g. centimeters, millimeters, inches or degrees). All types of publications besides narrative, systematic, or scoping reviews were eligible for inclusion. No limits were set on the number of included patients. The defined exclusion criteria were: narrative and systematic reviews, articles not on prominent ears, articles where the full text could not be found, and articles that measured outcomes solely subjectively (e.g. only containing narrative descriptions of patients and/or physicians’ opinions or subjective assessments measured on a Likert scale).

### Study selection and data collection

For the literature searched from inception until the 8^th^ of December 2022, two authors (YYL) and (HAB) independently screened the titles and abstracts of all records for inclusion using Rayyan^17^ after removing duplicates. Subsequently, they screened the full texts of potentially eligible records using Endnote. Any controversies regarding article eligibility were discussed with the third and the fourth reviewer (ER and CB). Two authors (YYL and HAB) also independently extracted the data from included articles using a predefined form. Data were extracted on study characteristics (type, authors, and year of publication), patients (number, age, sex, and the number of treated prominent ears), treatment (type and duration), and objective outcome measures used, as well as the reported outcomes. For the literature search updated from the 8^th^ of December 2022 until the 24^th^ of April 2023, three authors (YYL, HAB, and EMR) independently screened the titles and abstracts of all records for inclusion.

### Quality assessment

We used the critical appraisal checklist for case series from the Joanna Briggs Institute to assess the quality of the included studies.^18^ This checklist consists of ten questions on the methodological and reporting quality of a study that can each be answered with “yes,” “no,” “unclear” or “not applicable (n/a).” As this checklist does not provide a way of summarizing the quality of each study, answers to each question were appraised individually.

## Results

### Study selection

**Figure 1:**
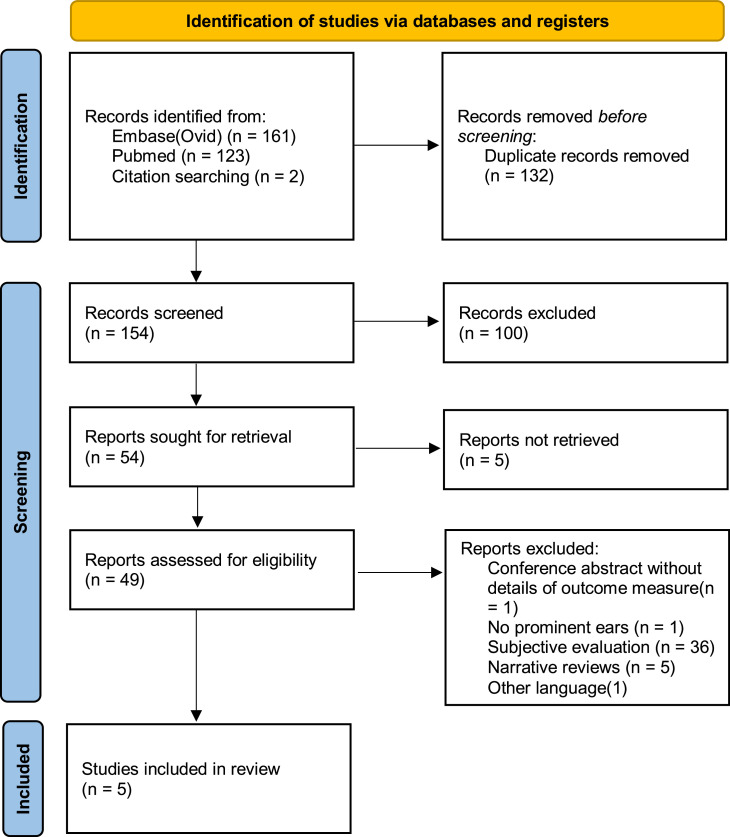
PRISMA 2020 flow diagram for new systematic reviews which included searches of databases, registers and other sources.

A literature search was performed in December 2022 and updated in April 2023, yielding 286 studies from two databases and citation searching, of which 132 were duplicates. Of the remaining 154 articles, 100 were excluded based on their titles and abstracts. Five articles could not be retrieved. Forty-four articles were excluded after full-text screening. The remaining five articles^3^^,^^9^^,^^19^, ^20^, ^21^ met the inclusion criteria. Studies were mainly excluded during full-text review due to subjective outcome assessments. The full search and screening process is also illustrated in Figure 1.

*From:* Page MJ, McKenzie JE, Bossuyt PM, Boutron I, Hoffmann TC, Mulrow CD, et al. The PRISMA 2020 statement: an updated guideline for reporting systematic reviews. BMJ 2021;372:n71. doi: 10.1136/bmj.n71. For more information, visit: http://www.prisma-statement.org/.

### Study and patient characteristics

All included studies were case series and included patients with prominent ears. Three studies^19^, ^20^, ^21^ also included other ear deformities or malformations. For these reviews, only data on patients with prominent ears was used. The included studies reported on a combined total of 208 patients. Of these 208 patients, 131 patients with 227 prominent ears completed the treatment. Seventy-seven patients were lost to follow-up in the studies by Van Wijk et al.^3^ and Sorribes et al.^9^ combined. The ages of the included patients were between three days and 5.5 years. Three studies used wire-shaped splints,^3^^,^^20^^,^^21^ one study used clip-shaped splints combined with silicon tape,^9^ and one study used the EarWell system^19^ [Table 1].

**Table 1:** Study characteristics of the prominent ears patients of included studies

Author	Year	Study type	Patients number	Ears number	Sex	Age, Mean ± SD	Treatment types	Treatments duration	Assessment tools	Evaluated parameters
Chen^19^	2021	RCS	? (?)	3 (3)	?	?	EarWell	25 ∼ 35 days	?	Ear length and breadth
Van Wijk^3^	2012	PCS	132 (81)	209 (161)	?	8.8 ± 5.6 weeks	Wire	?	Ruler	HMD
Sorribes^9^	2002	PCS	70 (44)	140 (56)	Male: 36 (23)	2.2 ± ? years	Clip + tape	1 ∼ 10 months	Thickness gauge	Pinna thickness
					Female:34 (21)				Ruler	HMD
									Tension gauge	Cartilage stiffness
Tan^20^	1997	RCS	4 (4)	8 (8)	?	9 weeks	Wire	5.5 ∼ 10 weeks	?	HMD
Tan^21^	1994	RCS	2 (2)	2 (2)	Male: 1 (1)					
Female: 1 (1)	2.5 ± 0.7 months	Wire	6 and 10 weeks	?	HMD					

PCS Prospective case series.

RCS Retrospective case series.

? Data missed.

Data within parentheses are about patients who finished treatments.

HMD Helix Mastoid Distance.

### Outcome measures

Four studies measured the HMD pretreatment and posttreatment^3^^,^^9^^,^^20^^,^^21^. Tan et al.^20^^,^^21^ reported the measurement results for each of the patients (n=4 and n=2, respectively). One study also measured pinna thickness and cartilage stiffness,^9^ and another measured the length and width of the ear. Due to the differences in used techniques and definitions, the measurements and outcomes will be discussed separately.

### Helix Mastoid Distance (HMD)

All studies that measured HMD used photographs for the measurements and summarized outcomes into subjectively defined groups (i.e. good, moderate, and poor results). All studies used different definitions for the specific measurement technique (or did not specify how the measurement was made) and the grading categories [Table 2].

**Table 2:** Summarization of HMD measurement methods

Author	Year	Treatment types	Parameters	Definitions	Data before treatment	Data after treatment	Results, number of patients	Explanations
Van Wijk^3^	2012	Clip + tape	HMD	The widest mastoid-helical distance	17.9 ± 3.1 mm	Good: 15.4 mm	Good: 23	The mean HMD was −2.5 mm for the “Good” category, +0.95 mm for the “Fair” category, and +3.8 mm for the “Poor” category
						Fair: 28.85 mm	Fair: 29	
						Poor: 21.7 mm	Poor: 29	
Sorribes^9^	2002	Wire	HMD	measured at four positions on the helix:	?	?	Good: 15 (19) Fair: 23 (31) Poor: 6 (6)	A reduction in HMD of 6-10 mm as “Good,” 3-5 mm as “Fair,” and 1-2 mm or less as “Poor.” Data outside the “()” are outcome measures of patients Data inside the “()” are outcome measures of auricles
				1. Superior point				
				2. Superior line				
				3. Frankfort horizontal line				
				4. Conchal line				
Tan^20^	1997	Wire	HMD	?	17.6 ± 3.2 mm*	13.1 ± 2.9 mm*	Good: 6 Poor: 2	Normal: HMD smaller than 15 mm. Good: HMD not being bigger than 15 mm and the HMD improving by more than 4 mm. Poor: HMD did not improve more than 4 mm
Tan^21^	1994	Wire	HMD	?	42 ± 9 mm*	26 ± 15 mm*	Excellent: 1 Improved (slight): 1	Excellent: an improvement of the HMD from 35 to 15 mm Improved (slight): an improvement of the HMD from 48 to 36 mm

⁎ Calculated from the raw data.

### Pinna thickness and cartilage stiffness

Sorribes et al.^9^ also measured pinna thickness and cartilage stiffness. Pinna thickness was defined as the thickness between the helix and the concha at the top of the auricle and measured with a thickness gauge. Cartilage stiffness was defined as the elastic tension of prominent ear cartilage when forced to form the antihelix. This parameter was measured by a dial tension gauge (gram force, ±5), which shows the tension on the scale when the antihelix is maximally folded by pressing the instrument arm toward the lateral part of the auricle. No statistical differences, however, were found in treatment outcomes between different degrees of thickness and tension.

### Ear length and width

Chen et al.^19^ measured ear length as the distance between the supaurale and subaurale landmarks and ear width as the distance between preaurale and postaurale landmarks. Outcome data for prominent ears was not reported.

### Study quality

Two studies scored “yes” on measuring the condition in a standard way by defining the condition in a valid way and consecutively including patients. Three studies scored “no” on clear reporting of the demographics of the participants in the study. Four studies scored “unclear” on clear reporting of the outcomes, frequently due to missing clear definitions describing the ways of objective measurements. The article by Chen et al.^19^ scored “no” on clear reporting, considering data on prominent ear patients were missing. The used critical appraisal can be found in Table 3.

**Table 3:** Critical appraisal

Author	Year	Q1	Q2	Q3	Q4	Q5	Q6	Q7	Q8	Q9	Q10
Chen^19^	2021	Unclear	No	Yes	Yes	Unclear	No	No	Unclear	No	Yes
Van Wijk^3^	2012	Unclear	Yes	Unclear	Yes	Yes	Yes	No	Unclear	Unclear	Yes
Sorribes^9^	2002	Yes	Yes	Yes	Yes	No	No	Yes	Yes	No	Yes
Tan^20^	1997	Yes	Unclear	Yes	Yes	Yes	No	Unclear	Unclear	No	N/A
Tan^21^	1994	Yes	Unclear	Yes	No	No	Yes	Yes	Unclear	No	N/A

## Discussion

This review summarized objective measurements used to assess outcomes of nonsurgical treatment of prominent ears. We found that the HMD was used most frequently, although ear length and width, ear thickness, as well as cartilage stiffness were also reported in individual studies. Despite the fact that the results of most of the studies were based on an objective measure, there was significant heterogeneity in how these parameters were measured and how results were subsequently grouped.

Due to the diagnostic definitions and anatomical characteristics of prominent ears, HMD is one of the most important aesthetic parameters for assessing the outcome of prominent ear treatment.^14^ Sorribes et al.^9^ measured HMD at four positions, whereas Van Wijk et al.^3^ and Tan et al.^20^^,^^21^ measured it at only one position. A recent review article on prominent ears and their treatment methods suggested measuring HMD along the entire helical rim, as the distance varies according to the measurement point.^22^ For example, in a cohort study of 102 patients without prominent ears, compared to 44 patients with prominent ears, protrusion was defined as an HMD of >21.55 mm in boys and >17.5 mm in girls at a point cranial to the middle of the helix, and >20 mm for boys and >15.5 mm for girls at a point caudal from the middle of the helix.^14^ As the reduction in HMD also varies depending on the point of measurement,^9^ measuring it at multiple points to fully reflect the effect of treatment seems necessary. However, even though measured as a continuous variable, all authors grouped the HMD into subjective outcome categories, which did not correspond across studies—a good result in the study by Van Wijk et al.^3^ or Tan et al.^20^ would have been scored as fair or poor by Sorribes et al.^9^ There is currently no consensus on the definition of good, fair or poor results,^6^ especially their correlation to the degree of HMD reduction postoperatively. Efforts have been made to provide HMD measurement methods and data on adult counterparts. However, these methods often use their own definitions and lack supporting evidence for their measurement techniques. The lack of a unified standard for ear shape not only leads to a lack of awareness of the exact prevalence of protruding ears but also creates a blurred boundary between protruding ears and normal ears, making it difficult to accurately assess the efficacy of various treatments.^23^ Further research is needed to summarize anthropometric measures of HMD in a normal population, to define normal protrusion, as well as the degree of asymmetry in normal populations so that surgical and nonsurgical results may be compared with these standards.

Few studies evaluate otoplasty outcomes by transforming continuous variables into categorical variables. Usually, categorical variables, such as scales, are generally used to record the operator's or patient's subjective aesthetic perception,^6^ whereas pretreatment and posttreatment changes in a continuous variable such as HMD are used to compare various treatment outcomes.^24^^,^^25^ Combining the two methods may not yield either an objective assessment of postoperative change or a subjective aesthetic outcome.

The other outcome parameters found in the current review were potentially less relevant for assessing the outcomes of the treatment of prominent ears, whereas other parameters generally considered important were not used at all. Although a lack of mechanical support of ear cartilage is a major cause for prominent ears, Sorribes et al.^9^ found no differences in cartilage stiffness or pinna thickness between outcome groups. Furthermore, ear length and width are the more commonly used parameters to reflect the size of the ear,^22^ and due to reporting limitations, it was unclear how Chen et al.^19^ applied these parameters to prominent ear treatment outcomes. Conversely, other parameters that are also aesthetically important for prominent ears, like auriculocephalic angle^26^ and symmetry,^27^ were not used by any studies included in this review. Geometrically speaking, the auriculocephalic angle does not vary depending on ear size or facial proportions and is more stable than the HMD. It seems particularly suitable for providing a clear criterion for the diagnosis of prominent ears, but accurately defining the two planes (one for the auricle and one for the mastoid) to determine the angle is difficult, and therefore, measurement error can be significant. The literature presenting data for this parameter often also lacks specific descriptions of the measurement^28^^,^^29^ or requires complex procedures like alginate molding.^26^^,^^30^ Moreover, none of the included studies assessed symmetry explicitly, another important aesthetic feature of normal ears.^23^

Most of these measurements are dependent on accurate landmarks to produce reproducible and comparable data. In biogeometric morphometry research, landmarks are anatomical points that provide homological biological and geometric information.^31^ These can be categorized into three types according to Bookstein (1) landmarks defined by the intersection of structures; (2) landmarks at the maxima of curvatures; and (3) landmarks defined by extreme value points of the structure, such as the longest and widest distance.^31^ Examples of type 1, 2, and 3 landmarks, respectively, include the outer canthus, used by Sorribes et al.,^9^ the most prominent point of the helix, which is commonly used to measure the HMD,^2^^,^^3^^,^^11^^,^^12^ as well as the preaurales, postaurales, supaurales, and subaurales, which are used for ear length and width.^19^ However, a review of the literature reported varying interpretations of these categories and subsequently varying classification as well as the minimal correlation between landmark type and the reproducibility of measurements.^32^ Using manual landmark-based measurements, therefore, hinders the uniform application of objective measurements.^15^^,^^33^ Digitalized automatic landmark recognition provides a possibility to objectively landmark an area using a computerized algorithm instead of manual placement and shows potential for improving objectivity.^34^^,^^35^ It has demonstrated the ability to accurately measure facial features,^36^ assign severity grades for cleft palate,^37^ and detect a large number of landmarks for cephalometric analysis.^38^^,^^39^ Although it is already a hot topic in ear recognition,^35^^,^^40^ this automatic landmark detection is yet to be applied to the evaluation of prominent ears. Automated algorithms based on three-dimensional imaging may also be used to accurately define the planes needed for measuring the auriculocephalic angle.^41^ Future research on prominent ears could focus on implementing automated, robust algorithms based on three-dimensional imaging for objective measurements of prominent ears.

This review has a few limitations. We only included literature in English. As prominent ears essentially affect the subjective psychological well-being of the patient, different socio-cultural environments may have different requirements for ideal aesthetics. The inclusion of studies from a wider range of linguistic and cultural backgrounds may provide more guidance in the selection of different automated auricular aesthetic parameters. Furthermore, this review only gives a general direction on possible automated auricular evaluation techniques; which automated techniques are suitable and provide clinically relevant information needs to be further refined by future studies.

## Conclusion

The use of objective measurements like the HMD is rarely seen in studies on nonsurgical treatment of prominent ears. Although HMD was the most frequent measurement used, all studies that described this measurement used different definitions to classify results. Furthermore, all studies relied on subjectively defined landmarks, which were heterogeneous. Objective landmark definitions are necessary to assess outcomes objectively and compare treatment results across studies. Three-dimensional automated algorithms could provide a solution for measuring and defining these landmarks, as well as the planes needed for accurate measurements. This would, in turn, aid the objective measurement of nonsurgical treatment outcomes of prominent ears.

## Ethical Approval

Not required.

## Funding

None.

## Declaration of Competing Interest

None.

## Appendix Supplementary materials

Image, application 1
