# Supplementary material for: Objective evaluation of nonsurgical treatment of prominent ears: A systematic review
Source: JPRAS Open. 2023 Jul 22;38:14–24. doi: 10.1016/j.jpra.2023.07.002 (PMC10491642; doi:10.1016/j.jpra.2023.07.002)
Supplement: Supplementary file 1 [file mmc1.docx]

*Objective evaluation of non-surgical treatment of prominent ears: a systematic review.*

8-12-2022:

| Databases: |  |  |
| --- | --- | --- |
| PubMed, Embase (Ovid) | Before deduplication | After deduplication |
| Total | 245 | 144 |

PUBMED

115 results:

("Ear"[Mesh] OR "Ear Auricle"[Mesh] OR "Ear, External"[Mesh] OR ear[Title/Abstract] OR ears[Title/Abstract])

AND

(deformity[Title/Abstract] OR malformation[Title/Abstract] OR deformities[Title/Abstract] OR malformations[Title/Abstract] OR protruding[Title/Abstract] OR protrusion[Title/Abstract] OR prominent[Title/Abstract])

AND

(non-surgical[Title/Abstract] OR splinting[Title/Abstract] OR molding[Title/Abstract])

EMBASE (OVID):

Database(s): Embase Classic+Embase 1947 to 2022 December 07
Search Strategy:

| **#** | **Searches** | **Results** |
| --- | --- | --- |
| 1 | (ear or ears or ear auricle or ear auricles or ear external).ti,ab,kf. | 161407 |
| 2 | external ear/ or ear/ or auricle/ | 43787 |
| 3 | 1 or 2 | 171554 |
| 4 | (deformity or malformation or deformities or malformations or protruding or protrusion or prominent).ti,ab,kf. | 516611 |
| 5 | (non-surgical or splinting or molding).ti,ab,kf. | 34461 |
| 6 | 3 and 4 and 5 | 130 |

UPDATE 8-12-2022 t/m 24-4-2023

**24-4-2023:**

| Databases: | Results update: | Results update: |
| --- | --- | --- |
| PubMed, Embase (Ovid) | Before deduplication | After deduplication |
| Total | 39 | 8 |

PUBMED: 8 results:

("Ear"[Mesh] OR "Ear Auricle"[Mesh] OR "Ear, External"[Mesh] OR ear[Title/Abstract] OR ears[Title/Abstract])

AND

(deformity[Title/Abstract] OR malformation[Title/Abstract] OR deformities[Title/Abstract] OR malformations[Title/Abstract] OR protruding[Title/Abstract] OR protrusion[Title/Abstract] OR prominent[Title/Abstract])

AND

(non-surgical[Title/Abstract] OR splinting[Title/Abstract] OR molding[Title/Abstract])

AND ("2022/12/08"[Date - Publication] : "2023/04/24"[Date - Publication])

EMBASE (OVID):

Database(s): **Embase Classic+Embase**1947 to 2023 April 21
Search Strategy:

| **#** | **Searches** | **Results** |
| --- | --- | --- |
| 1 | (ear or ears or ear auricle or ear auricles or ear external).ti,ab,kf. | 165737 |
| 2 | external ear/ or ear/ or auricle/ | 45312 |
| 3 | 1 or 2 | 176169 |
| 4 | (deformity or malformation or deformities or malformations or protruding or protrusion or prominent).ti,ab,kf. | 535990 |
| 5 | (non-surgical or splinting or molding).ti,ab,kf. | 36080 |
| 6 | 3 and 4 and 5 | 142 |
| 7 | limit 6 to yr="2022 -Current" | 31 |
